# Shear Stress Transmission Model for the Flagellar Rotary Motor

**DOI:** 10.3390/ijms9091595

**Published:** 2008-09-01

**Authors:** Toshio Mitsui, Hiroyuki Ohshima

**Affiliations:** 1 Nakasuji-Yamate 3-6-24, Takarazuka, 665–0875, Japan; 2 Faculty of Pharmaceutical Sciences, Tokyo University of Science, 2641 Yamazaki, Noda, Chiba 278–8510, Japan. E-Mail: ohshima@rs.noda.tus.ac.jp

**Keywords:** Flagellar rotary motor, model for flagellar motor, proton-induced electric field in membrane, permanent electric dipole, viscoelasticity, motor driven by shear force, rotation velocity vs. proton deriving force, torque-velocity relation

## Abstract

Most bacteria that swim are propelled by flagellar filaments, which are driven by a rotary motor powered by proton flux. The mechanism of the flagellar motor is discussed by reforming the model proposed by the present authors in 2005. It is shown that the mean strength of Coulomb field produced by a proton passing the channel is very strong in the Mot assembly so that the Mot assembly can be a shear force generator and induce the flagellar rotation. The model gives clear calculation results in agreement with experimental observations, e g., for the charasteristic torque-velocity relationship of the flagellar rotation.

## 1. Introduction

Most bacteria that swim are propelled by flagellar filaments, which are driven by a rotary motor embedded in the cell wall and the cytoplasmic membrane. The motor is powered by proton flux (or in some species, sodium ion flux). In 2000, Berg [1] summarized the constraints that models for the flagellar rotary motor should satisfy, and noted that the existing models did not satisfy those constraints. In 2005, the present authors proposed a model for the flagellar motor [2] from a viewpoint quite different from other models. Protein molecules are structurally polar and thus there should be an interaction between an electric field and the mechanical deformation of protein molecules, as usually discussed in material physics (cf, for instance, [3]). This means that theoretically the flagellar rotary motor should be treated as a four-variable system, i. e., as a system in which strain, stress, electric field and polarization are interacting with each other. The models proposed by other authors seem to have neglected this viewpoint. It was shown in [2] that the electric Coulomb field produced by a proton passing through the channel is so strong in Mot assemblies that it can induce a significant shear stress in the stator. If the layer between the stator and rotor is viscoelastic, the shear stress propagates through the viscoelastic layer resulting in rotation of flagella. This new model gave clear calculation results in agreement with experimental observations.

Naturally, the final goal to understand the mechanism of the flagellar motor will be an explanation based upon more detailed structures of composed protein molecules, as suggested by the model proposed by Xing *et al.* [4] in 2006. Many protons (about 1000) are, however, required for one revolution of flagellar rotation, which suggests a possibility that the mechanism of the flagellar rotation is physical rather than chemically specific. Accordingly, it seemed worthwhile to introduce that such model as [2] can give systematic explanation of the experimental data. The thesis [2], however, contains several immature discussions. In the present paper, we have reformed the model and tried to make the description more readable.

In Section 2, the simplified structure of the flagellar rotary motor is illustrated and major experimental results to be explained by the model are summarized. Basic ideas of the new model are outlined in Section 3. The model is formulated in Section 4. Theoretical predictions are compared with experimental observations in Section 5. Results are summarized and discussed in Section 6.

## 2. Objects to be discussed

### 2.1. Structure of the flagellar rotary motor

**Figure 1.**
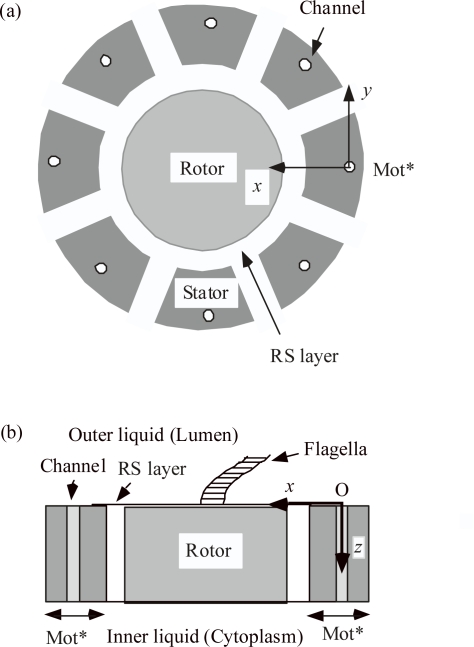
Simplified structure of flagellar rotary motor. (a) Top view, (b) Vertical section. Actually Mot* has two proton channels [6], but, for simplicity, one channel is depicted in which a proton pair passes (see text).

Figure 1 shows a simplified structure of the flagellar rotary motor. The motor has a diameter of approximately 45 nm [1] and is surrounded by a membrane with an averaged thickness of approximately 7 nm. The motor consists of Rotor, Stator and the intermediate layer between them, which is hereafter called the RS layer. The stator consists of complexes of Mot A and Mot B proteins. The complex is symbolized as Mot* and the Stator is assumed to have eight Mot*s [5], as illustrated in Figure 1(a). Each Mot* has two proton channels [6]. Two protons seem to pass practically simultaneously through the two channels [6]. For simplicity, we suppose that two protons simultaneously pass through the combined single channel shown in Figure 1. The proton pair is called 2e, where e represents the charge of proton.

Under normal conditions, the proton channels conduct protons from the lumen to the cytoplasm. The electrolytes in the lumen and the cytoplasm are referred to as the outer liquid and the inner liquid, respectively. A flagellum has its root in the center of the rotor, and rotates at the speed of, for example, 10 or 100 Hz, depending upon the viscous resistance of the outer liquid. The Mot* shown at the extreme right in Figure 1 will be discussed as a representative of Mot*s. The origin of the coordinate system is set at the outer surface of the single channel situated at its center, the *x* axis is directed toward the axis of the rotor, the *z* axis is perpendicular to the membrane, directing inward to the cytoplasm, and the *y* axis is orthogonal to the *x* and *z* axes, as shown in Figure 1.

### 2.2. Major experimental results to be explained by the model

Experimental observations on the flagellar rotary motor to be explained by the model are summarized as follows:

(1) One revolution of the flagellar rotation consists of a constant number of steps at low speed [1].
(2) The flagellar rotation velocity *ω* is proportional to the transmembrane potential difference at low speed [1].
(3a) The torque for the cell to rotate flagella is practically constant independent of rotational velocity *ω* in the range from *ω* = 0 to a critical value *ω*_cr_ (approximately 200 Hz at room temperature) and then decreases sharply (cf, Figure 8) [1].
(3b) When *ω* is smaller than *ω*_cr_, the torque varies little with temperature [1].
(3c) The critical velocity *ω*_cr_ shifts to lower speed at lower temperatures [1] (cf. Figure 8).
(3d) Where *ω* is larger than *ω*_cr_, the torque declines more steeply at lower temperatures [1] (cf. Figure 8).
(4) There are experimental observations that the flagellar rotate in the same direction when the direction of the proton passage is reversed (for references, cf. Section 5.2).
(5) The cell produces constant torque to rotate a flagellum even when the cell is rotated relative to the flagellum by external forces (for references, cf. Section 5.3).
(6) The cell has a switch that reverses the sense of the flagelllar rotation with the same absolute value of torque for chemotaxis (for references, cf. Section 5.4).

## 3. Basic ideas of the new model

The basic ideas of the new model, which are quite different from other models, are explained in this section before getting into details.

### 3.1. Mot* is assumed to be a shear force generator

Our basic assumption is that the electric field produced by protons plays an important role in the flagellar rotation. As the first trial model, the proton channel was assumed to be tilted to the *z*-axis on the *y–z* plane. Then the proton passage in the channel will give Rotor a torque if the surface of Rotor has a suitable charge distribution. This model, however, did not explain the experimental data and was discarded. Then, it is assumed that the proton channel is perpendicular to the membrane as shown in Figure 1(b) and Mot* has an electric polarization along the *y*-axis so that Mot* acts as a shear force generator. This model successfully explained experimental observations.

**Figure 2.**
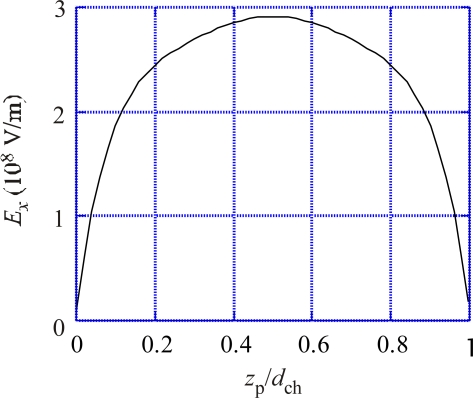
*E_x_* vs. *z*_p_/*d*_ch_. *E_x_*: volume-averaged *x* component of the electric field produced by the proton pair. *z*_p_: *z* coordinate of the proton pair. *d*_ch_ : 7 nm, the channel length.

In order to obtain rough idea on the strength of the field produced during the proton passage in Mot*, a dielectric membrane is considered as described in Appendix, where Coulomb field is calculated by the method of images as a function of *z*_p_, the proton-pair position on the *z* axis (the normal of the dielectric membrane). Since the distance between the channel and the Rotor surface is uncertain, a cylinder of radius 2.5 nm is considered as an example. The *x* component of the field is averaged over the half the cylinder of *x* > 0 and the average is denoted as *E_x_*. The mean *z* component of the field is zero since the membrane is sandwiched by conductors. Also the volume-averaged *E_y_* is zero due to the symmetry of the dielectric membrane. Hence, only the *x* component of the field is considered. Figure 2 shows *E_x_* as a function of *z*_p_/*d*_ch_, where *d*_ch_ is the channel length. Thus *z*_p_/*d*_ch_ stands for the relative position of the proton pair in the channel. The proton pair sits at the outside end of the channel when *z_p_*/*d*_ch_ = 0 and at the inside end when *z*_p_/*d*_ch_ = 1. The calculation was made by setting *d*_ch_ equal to 7 nm. The order of magnitude of *E_x_*(*z*_p_) around the middle of *z*_p_ (3.5 nm) is 3×10^8^ V/m. Note that the breakdown field strength of bulk paraffin is about 10^7^ V/m, but the field can be stronger than 5×10^7^ V/m in a synthetic polymer film of thickness 50 mm (for instance, cf. [7]). It seems possible that the breakdown fields are stronger than 5×10^7^ V/m in microscopic systems. Since the field of 3×10^8^ V/m is very strong, it is plausible that it can produce significant effects on Mot* structures if Mot* has charged parts in it. Note that Figure 2 is to give an idea on the strength of the field and actual field in Mot* should have more complex characteristics, e. g., it is asymmetric since the membrane structure is polar. For simplicity, however, the same notation *E_x_* will be used for the *x* component of the actual field in Mot*in the following discussion.

**Figure 3.**
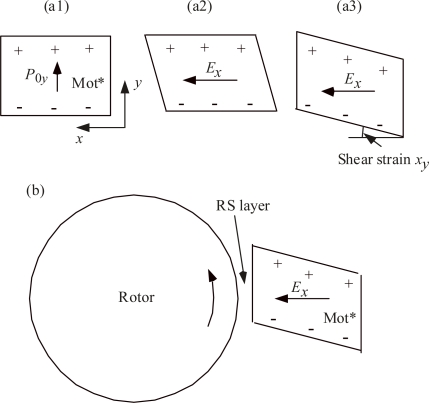
Interaction between *P*_0*y*_ and *E_x_*, shear strain *x_y_* in Mot*, and the effect of *x_y_* on Rotor. (a1) The rectangle corresponds to Mot*. The electric charges + and − are due to Mot* polarization *P*_0*y*_. (a2) Shear deformation due to the shifts of the charged part, which are caused by the electric field *E_x_*, when the basal edge of the rectangle is fixed. (a3) Shear deformation when the right-hand edge of the rectangle is fixed. The shear strain *x_y_* corresponds to the angle indicated. (b) Rotation of the rotor due to the deformation of Mot* (a3).

Protein molecules are structurally polar and generally they have large permanent electric dipole moments [8, 9]. For instance, G-actin has the dipole moment of 600 Debye and flagellin about 800 Debye [9]. As shown in Figure 2 of [10], the MotA complex consists of 4 copies and MotB consists of 2 copies in Mot*. All the copies are expected to have permanent electric dipoles. We call the polarization corresponding to the vector sum of their dipoles as Mot* polarization, and denote its *y* component as *P*_0*y*_. Interaction between *P*_0*y*_ and *E_x_* will cause a shear stress and strain in Mot*. Figure 3(a1) indicates Mot* by the rectangle arrow and electric charges due to *P*_0*y*_ by + and −. The arrow in (a2) indicates the field *E_x_*. The positive charges of Mot* will move to left and the negative charges move to right if Mot* is free to deform. Thus the rectangle will deform as indicated in (a2) when the lower side of the Mot is fixed. The deformation will take place as shown in (a3) when the right-hand side of Mot* is fixed, and will tend to rotate Rotor through RS layer as shown in Figure 3(b). The angle indicated in Figure 3(a3) is the shear strain *x_y_*. The shear stress which produces *x_y_* is denoted as *X_y_*, which is approximately proportional to *P*_0*y*_ and *E_x_*(*z*_p_), i.e. to *P_0y_E_x_*(*z*_p_) when the proton pair 2e sit at *z*_p_. The stress *X_y_*(*z*_p_) is written by

(1) $$X_{y}(z_{\text{p}})=aP_{0y}E_{x}(z_{\text{p}})$$

where *a* is a constant determined by the mechanical properties and shape of Mot*. Here, Mot* is embedded in the membrane as a part of the flagellar motor system, and the stress *X_y_*(*z*_p_) tends to transmit into its surroundings (cf. for instance [11]). As discussed in the next section, RS layer is viscoelastic and a portion of the stress *X_y_*(*z*_p_) will be transmitted into RS layer. The way of the transmission will be discussed in Section 3.3.

Let us quantitatively examine the possibility that Rotor rotates by the strain *x_y_*. According to Figure 1 of [1], the diameter of Mot A is comparable to the radius of Rotor. Hence the strain *x_y_* will be of the same order of magnitude as Rotor rotation angle. As an example for large piezoelectric strain, let us consider the ferroelectric crystal, KH_2_PO_4_ [12]. In this crystal, *x_y_* = *d*_36_ *E*_z_ and its maximum *d*_36_ is 1.4×10^−9^ m/V at −150 °C. If *E*_z_ is 10^8^ V/m, *x_y_* becomes 0.14 radian. If this value is adopted as *x_y_* of Mot*, 2*π* /0.14 = 45 proton pairs are required for one revolution. If *d*_36_ is one tenth of 1.4×10^−9^ m/V, 2*π*/0.014 = 450 proton pairs are required. It is known that about 1000 protons are required per revolution of Rotor [1]. The cited *d*_36_ is the value at the temperature where KH_2_PO_4_ does not have the permanent polarization. In the presence of the permanent polarization, the shear strain may become larger. If Mot* is designed as a shear force generator having the permanent polarization, it will be relatively easy to produce shear stress that is large enough to produce the flagellar rotation.

### 3.2. The RS layer is viscoelastic

The flagellar motor is embedded in liquid membranes. It is assumed that the RS layer consists of lipid membranes. According to [13], lipid membranes are generally viscoelastic, that is, they behave like a viscous liquid as well as an elastic medium in which shear stress can propagate. Here, by the terminology of viscoelasticity, we expect the characteristics of RS layer that the shear stress can transmit in it and that the layer acts as lubricant or (cylindrical) ball bearings for the rotation of Rotor. It seems permissible to assume that RS layer has a finite thickness though it is thin, and thus can have such characteristics.

Concerning the nature of the lipid membrane, an observation by Nakayama *et al.* [14] seems interesting. They extracted lipids from the cytoplasmic membrane of *E. coli* cells and studied a relation between the growth temperature and the solid-liquid phase transition in the lipid membrane with X-ray diffraction method. They found that cytoplasmic membranes of the cells grown at 17, 27 and 37 °C exhibited the lipid solid-liquid phase transitions at 10, 15 and 28.5 °C, respectively. That is, the transition temperatures were below the growth temperatures by 7, 12 and 8.5 °C, respectively, and thus the lipid parts of the cytoplasmic membranes were always in the liquid phase. Nakayama *et al.* [14], who carried out also chemical assays, and found that the molar ratio of the saturated to unsaturated fatty acids increases in phospholipids extracted from cytoplasmic membranes as the growth temperature increases. It seems plausible that E. coli cells adjust the molar ratio of the saturated to unsaturated fatty acids in order that the viscoelastic properties of lipid layer remain constant irrespective of the growth temperature.

**Figure 4.**
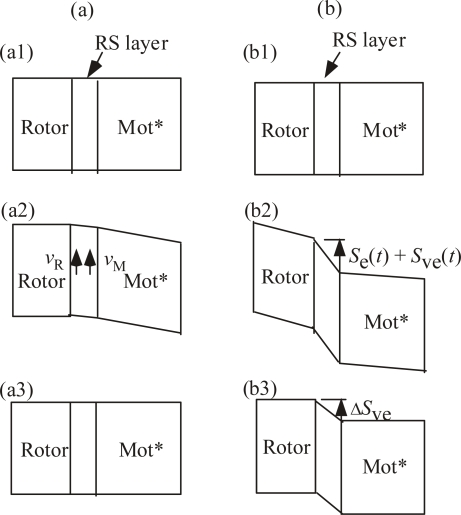
Response of the system against the shear force transmission. A part of Rotor, RS layer and Mot* are represented by rectangles (or parallelograms after deformation). (a) Simple viscous response. (b) Viscoelastic response. (a1), (b1): Before the shear stress takes place. (a2), (b2) During the shear stress is passing. (a3), (b3) After the shear stress has passed. Remanent displacement Δ*S*_ve_ of Rotor is left in (c3).

Figure 4 illustrates responses of the system against the shear force transmission, when RS layer is simply viscous (a), and when the system is viscoelastic (b). A part of Rotor, RS layer and Mot* are represented by rectangles (or parallelograms after deformation). Figures (a1) and (b1) show the states before the shear stress takes place. (a2) and (b2) show the states when the shear stress is passing. The shift of the boundary between Rotor and RS layer corresponds to the step rotation of Rotor and the deviation of the Rotor shape from rectangle (b2) indicates the propagation of the shear stress in Rotor. (a3) and (b3) show the states after the shear stress has passed. In Figure (a2), the arrows *ν*_M_ indicates the viscous stream following the movement of the edge of Mot*. As usually occurs in a viscous fluid (cf. for instance [15]), *ν*_M_ causes a stream *ν*_R_ near Rotor, causing movement *S*_v_(*t*) of Rotor. Positive *ν*_M_ will induce positive *S*_v_(*t*), while negative *ν*_M_ negative *S*_v_(*t*), and Rotor will returns to the original position when the shear stress passes as indicated in (a3). In the case of (b), the shear stresses transmitted into RS layer due to the viscoelastic property of RS layer. In addition to the direct effect of viscosity as illustrated in (a2), two effects of the transmitted stress are expected as illustrated in (b2). One is the effect of the elastic stress itself that causes movement *S*_e_(*t*) and elastic deformation of Rotor (b2), but these changes return to zero after the shear stress passes (b3). The second is the effect that the shear stress causes viscoelastic movement *S*_ve_(*t*) and thus rotation of Rotor (b2). The time derivative of *S*_ve_(*t*) is proportional to the shear stress *F_y_*(*t*) as usually treated as Maxwell’s model of viscoelastic systems [16, 17], and leaves the step rotation Δ*S*_ve_ after an impulsive stress has passed (b3), as discussed mathematically in Section 4-1a.

### 3.3. Expression for the shear force transmitted into the RS layer

The transmembrane electrochemical potential difference Δ*Ψ* is defined by

(2) $$\Delta \Psi \;=\;\Psi_{\text{in}}-\Psi_{\text{out}}$$

where *Ψ*_in_ and *Ψ*_out_ are the electrochemical potentials in the inner and outer liquids, respectively. The absolute value of the proton velocity is denoted as *ν*_p_, The frictional resistance against the proton motion in the channel is assumed constant as generally done. Then *ν*_p_ is proportional to |Δ*Ψ*| :

(3) $$\nu_{\text{p}}=A|\Delta \Psi |$$

where *A* is a constant. In this section the case of Δ*Ψ* > 0 is considered (the case of Δ*Ψ* < 0 is discussed in Section 4.2.). Then the proton position *z*_p_ is given as a function of time *t* by

(4) $$Z_{\text{p}}=\nu_{\text{p}}t$$

where *t* changes from 0 to *d*_ch_/*ν*_p_. The strength of the stress flow transmitted into RS layer is denoted as *F_y_*(*t*). It seems reasonable to assume that *F_y_*(*t*) is proportional to the stress *X_y_*(*ν*_p_*t*) produced in Mot* given by Eq. 1 so that *F_y_*(*t*) is a function of *ν*_p_, expressed by

(5) $$F_{y}(t)=X_{y}(\nu_{\text{P}}t)\text{f(}\nu_{\text{p}}\text{)}\;=\;aP_{0y}E_{x}(z_{\text{p}})\text{f(}\nu_{\text{p}}\text{)}$$

where f(*ν*_p_) is a function of *ν*_p_ to be determined. The energy input to the RS layer during d*t* is denoted as *W*d*t*. Then, *W* is given by

(6) $$W(t)=F_{y}{\left(t\right)}^{2}/(2c_{\text{RS}})$$

where *c*_RS_ is an elastic constant of RS layer. The total energy input associated with the proton pair passage is denoted as *I_W_*:

(7) $$I_{W}=\int Wdt$$

From Eqs, 5 and 6, *I_W_* becomes

(8) $$I_{W}=\{{\left(aP_{0y}\right)}^{2}/(2c_{\text{RS}})\}\;\int E_{x}{\left(\nu_{\text{p}}t\right)}^{2}\;\text{f(}\nu_{\text{p}}{\text{)}}^{2}\text{d}t$$

where integration is done from 0 to *d*_ch_/*ν*_p_. Since the proton velocity *ν*_p_ is constant and d*t* is given by d*z*_p_/*ν*_p_, Eq. 8 can be rewritten as

(9) $$I_{W}=\{\text{f}{(\nu_{\text{p}}\text{)}}^{2}/\nu_{\text{p}}\}\;\{\;{\left(aP_{0y}\right)}^{2}/(2c_{\text{RS}})\}\;{\int E_{x}(z_{\text{p}})}^{2}\;\text{d}z_{\text{p}}$$

The energy *I_W_* should be proportional to the work done by the membrane during the proton pair passage and thus proportional to |Δ*Ψ*| and to *ν*_p_ by Eq. 3. The integral ∫*E_x_*(*z*_p_)^2^d*z*_p_ is a constant independent of *ν*_p_. Hence, f(*ν*_p_)^2^/*ν*_p_ should be proportional to *ν*_p_:

(10) $$\text{f(}\nu_{\text{p}}\text{)}\;=\;\gamma \nu_{\text{p}}$$

where *γ* is a constant. The value of *γ* is assumed to be independent of other parameters, e. g., *c*_RS._ This assumption means that Mot* is a constant shear force transmitter. The model based upon this postulation explains various experimental observations as seen below. Now Eq. 5 becomes

(11) $$F_{y}(t)=\gamma X_{y}(\nu_{\text{p}}t)\nu_{\text{p}}=a\gamma P_{0y}E_{x}(z_{\text{p}})\nu_{\text{p}}$$

Integration of the force of the force *F_y_*(*t*) is denoted as *I_y_*:

(12) $$I_{y}=\int F_{y}(t)\text{d}t$$

where the integration is made from 0 to *d*_ch_/*ν*_p_. Combining Eqs. 4, 11 and 12 gives

(13) $$I_{\text{y}}=a\gamma P_{0y}\int E_{x}(z_{\text{p}})\text{d}z_{\text{p}}$$

Accordingly, *I*_y_ does not depend upon *ν*_p_ nor Δ*Ψ*.

As will be discussed in Section 4.2, Eq. 11 will be modified in the case of Δ*Ψ* < 0 (cf. Eq. 59), but the value of *I_y_* will remain unchanged.

## 4. Theoretical predictions on flagellar rotation

### 4.1. Flagellar rotation velocity and torque

In this section, discussion is continued for the case of Δ*Ψ* > 0.

#### 4.1.a. Step size of Rotor rotation

Now we derive an expression of the step rotation of Rotor corresponding to Δ*S*_ev_ in Figure 4(b3). In Section 3.2, the effects of the shear force *F_y_*(*t*) are discussed qualitatively by dividing them into three responses: (1) direct viscous response, (2) direct elastic response, (3) viscoelastic response. The direct viscous response (1) does not leave the flagellar rotation as illustrated in Figure 4(a3). The response (2) is illustrated as *S*_e_(*t*) in Figure 4(b2). It is assumed that *S*_e_(*t*) obeys Hooke’s law, then

(14) $$S_{\text{e}}(t)=F_{y}(t)/c_{\text{RS}}$$

where *c*_RS_ is the same elastic constant of the RS layer as in Eq. 6. Since *S*_e_(*t*) becomes zero when *F_y_*(*t*) becomes zero as illustrated in Figure 4(b3), *S*_e_(*t*) does not contribute to the rotation of the Rotor.

The viscoelastic response (3) is illustrated by the displacement of the Rotor side, *S*_ve_(*t*), in Figure 4(b2). Following Maxwell’s model of viscoelastic systems [16, 17], the time-derivative of *S*_ve_(*t*) is proportional to the deriving force:

(15) $$\zeta \text{d}S_{\text{ve}}(t)/\text{d}t=F_{y}(t)$$

where is a constant. Let us denote the radius of Rotor as *r*_R_ and the rotation angle corresponding to the displacement of the Rotor side *S*_ve_(*t*) as (*t*). Then

(16) $$S_{\text{ve}}(t)=r_{\text{R}}\theta (t)$$

and Eq. 15 becomes

(17) $$\left(r_{\text{R}}\zeta )\text{d}\theta (t)/\text{d}t=F_{y}(t\right)$$

This equation means that there is a balance between the viscous resistance against the flagellar rotation and the shear force *F_y_*(*t*). The parameter *ζ* seems to depend sensitively upon the viscosity *η* of the outer fluid, in which the flagellar rotates, and to be approximately proportional to *η*. To make this point clear, a new parameter *b* is introduced, which is defined by elastic properties of the RS layer and dimensions of the RS layer and Rotor.

(18) $$b=r_{\text{R}}\theta /\eta$$

Then Eq. 17 becomes

(19) $$\left(b\eta )\text{d}\theta \;(t)/\text{d}t=F_{y}(t\right)$$

The step displacement of the the Rotor side due to one proton pair passage is indicated as Δ*S*_ve_ in Figure 4(b3). The corresponding variation of *θ* is denoted as Δ*θ*. Then, by integration of Eq. 19,

(20) $$\Delta \theta =(1/b\eta )\int F_{y}(t)\text{d}t$$

The integration is already denoted as *I_y_* by Eq. 12, and Eq. 20 can be written as

(21) $$\Delta \theta =I_{y}/(b\eta )$$

As mentioned in connection with Eq. 13, *I_y_* does not depend upon the transmembrane potential difference Δ*Ψ*, and thus the step size Δ*θ* does not depend upon Δ*Ψ*.

#### 4.1.b. Rotation velocity as a function of the transmembrane potential difference

The number of proton pairs passing through one rotary motor per unit time is denoted as *n* which is defined as its absolute value irrespective of the proton passing direction. This number *n* is expected to be proportional to the transmembrane electrochemical potential difference Δ*Ψ*defined by Eq. 2. Since *n* is defined as the absolute value,

(22) n=B|ΔΨ|

where *B* is a constant.

If the rotational velocity of the rotor is denoted as *ω* (radian/s), then, *ω* is given by the step rotation Δ*θ*:

(23) ω=nΔθ

By Eq. 21, *ω* is given by

(24) $$\omega =nI_{y}/(b\eta )$$

Then, with Eq. 22, Eq. 24 becomes

(25) ω=C|ΔΨ|

(26) $$\text{C}\;=\;BI_{y}/(b\eta )$$

#### 4.1.c. Energy balance and critical rotation velocity

In some theories, the energy used for the flagellar rotation is set equal to the proton-motive force proportional to Δ*Ψ*. In our model, on the other hand, the energy of the transmitted shear force is not totally absorbed in the RS layer, but its rest transmits into the interior of the Rotor, causing shear strain in it, as illustrated by the deformation of the Rotor rectangle in Figure 4(b2). Thus the flagellar rotation is not discussed by the balance of energies but by the balance of forces as was done by Eq. 17. In the case, however, where the viscosity of the outer liquid is low, the total transmitted energy is spent by the flagellar rotation and the balance of energies has to be considered instead of the balance of forces.

It seems plausible that Mot* is designed to produce the flagellar rotation with good efficiency so that the simple viscous flow *ν*_M_ in Figure 4(a2) becomes small. One of such designs seems to make the magnitude of *x_y_* of Mot* as small as possible, as will be discussed briefly in Section 5.2 citing [2]. Hence the energy dissipation due to *ν*_M_ is neglected in the following discussion.

For simplicity, *S*_ve_(*t*), *F_y_*(*t*) and *θ*(*t*) in Eqs. 15 and 16 are written as *S*_ve_, *F_y_* and *θ*, respectively below. The energy liberation by the Rotor rotation during d*t* is expressed by the work *F_y_*d*S*_ve_= *F_y_*(d*S*_ve_/d*t*)d*t*, and thus, by Eq. 15,

(27) $$F_{y}\text{d}S_{\text{ve}}=\{F_{y}^{2}/\zeta \}\text{d}t$$

Let the energy liberation during d*t* be *D*d*t*, then, by Eq. 18,

(28) $$D=r_{\text{R}}F_{y}^{2}/(b\eta )$$

Writing *W*(*t*) of Eq. 6 as *W*, the ratio *D*/*W* becomes

(29) $$D/W=2c_{\text{RS}}r_{\text{R}}/(b\eta )=(2c_{\text{RS}}r_{\text{R}}/b)/\eta$$

Since the energy dissipation *D* must be smaller than the energy input *W*:

(30) D/W<1

Or, by Eq. 29,

(31) $$(2c_{\text{RS}}r_{\text{R}}/b)<\eta$$

The critical viscosity *η*_cr_ is defined by

(32) $$\eta_{\text{cr}}=2c_{\text{RS}}r_{\text{R}}/b$$

By Eqs. 25 and 26, the corresponding critical rotation velocity *ω*_cr_ is given by

(33) $$\omega_{\text{cr}}=\{BI_{y}/(b\eta_{\text{cr}})\}\;|\Delta \Psi |$$

By combining with Eq. 32,

(34) $$\omega_{\text{cr}}=\{BI_{y}/(2c_{\text{RS}}r_{\text{R}})\}\;|\Delta \Psi |$$

By Eq. 32, Eq. 31 can be written as

(35) $$\eta >\eta_{\text{cr}}$$

When *η* > *η*_cr_, we have that *D* < *W* and the difference *W* - *D* will be transmitted into the interior of the Rotor, causing an elastic deformation, as illustrated by the deformation of the rectangular Rotor in Figure 4(b2), and thus there is no problem to discuss the kinetics by balance of forces as was done in Section 4.1a.

When *η* < *η*_cr_, all the input energy is used for the Rotor rotation. There is no balance of forces and the flagella seem to cause vortex in the outer fluid so that the Rotor does not rotate smoothly. Let us consider a statistical ensemble of flagellar rotary motors, and denote the statistically averaged rotation angle at *t* as θ*. An averaged shear force F_y_* is defined as if there is the same balance of forces as Eq. 19:

(36) $$F_{y}^{*}=(b\eta )\text{d}\;\;\theta^{*}/\text{d}t$$

If the mean energy dissipation during d*t* is denoted as D*dt, D* is given by

(37) $$D^{*}=F_{y}^{*}r_{\text{R}}\text{d}\;\theta^{*}/\text{d}t\;=b\eta r_{\text{R}}{\left(\text{d}\theta^{*}/\text{d}t\right)}^{2}$$

If this D* is set equal to the input energy *W* given by Eq. 6, then we have

(38) $$b\eta r_{\text{R}}{\left(\text{d}\theta^{*}/\text{d}t\right)}^{2}=F_{y}^{2}/(2c_{\text{RS}})$$

which is rewritten as

(39) $$\text{d}\theta^{*}/\text{d}t=F_{y}/{\left({2c}_{\text{RS}}b\eta r_{\text{R}}\right)}^{1/2}$$

Integration of d θ*/d*t* during the proton pair passage is denoted as Δθ*. By using *I_y_* of Eq. 12, we have

(40) $$\Delta \theta^{*}=I_{y}/{\left({2c}_{\text{RS}}b\eta r_{\text{R}}\right)}^{1/2}$$

By using *η*_cr_ (Eq. 32), θ* can be written as

(41) $$\Delta \theta^{*}=(I_{F}/b)/{\left(\eta_{\text{cr}}\eta \right)}^{1/2}$$

Note that Δθ* is equal to Δ*θ* (Eq. 21) when *η*= *η*_cr_. When *η*< *η*_cr_, analogously to Eq. 23, ω* is defined by

(42) $$\begin{array}{ll} \omega^{*}=n\Delta \theta^{*}, & \omega >\omega_{\text{cr}} \end{array}$$

By Eq. 41,

(43) $$\begin{array}{ll} \omega^{*}=n(I_{y}/b)/{\left(\eta_{\text{cr}}\eta \right)}^{1/2}, & \omega >\omega_{\text{cr}} \end{array}$$

By Eq. 22, ω* becomes

(44) $$\begin{array}{ll} \omega^{*}=B(I_{y}/b)|\Delta \Psi |\;\;/{\left(\eta_{\text{cr}}\eta \right)}^{1/2}, & \omega >\;\omega_{\text{cr}} \end{array}$$

#### 4.1.d. Torque as a function of rotation velocity

From Eqs 25, 26, 32 and 33, we have

(45) $$\begin{array}{ll} \omega /\omega_{\text{cr}}=\eta_{\text{cr}}/\eta , & \eta >\eta_{\text{cr}}, \end{array}$$

**Figure 5.**
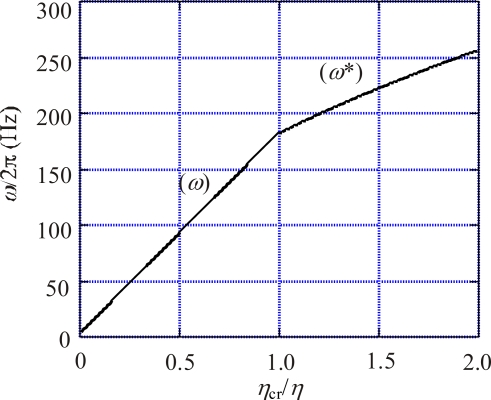
Rotation velocity *ω* /2*π* as a function of *η*_cr_/*η*, calculated by setting *ω*_cr_/2*π* = 180 Hz. (*ω*): *ω* calculated by Eq. 45. (*ω* *): upper limit of *ω* calculated by Eq. 48.

Figure 5 shows *ω* as a function of *η*_cr_/*η*, where we have put *ω*_cr_/2*π*= 180 Hz. From Eq. 45 we obtain the relation *ω* < *ω*_cr_ for *η* > *η*_cr_, and Eq. 45 can be rewritten as

(46) $$\begin{array}{ll} \eta \;\omega =\eta_{\text{cr}}\;\omega_{\text{cr}}, & \omega <\;\omega_{\text{cr}} \end{array}$$

The torque deriving the flagella is proportional to *η* and *ω*, and Eq. 46 mean that torque is constant independently of *ω* for *ω* < *ω*_cr_.

In the case of *η* < *η*_cr_, combining Eqs. 33 and 44 gives

(47) $$\begin{array}{ll} {\left(\omega^{*}/\omega_{\text{cr}}\right)}^{2}=\eta_{\text{cr}}/\eta , & \omega^{*}>\omega_{\text{cr}} \end{array}$$

or

(48) $$\begin{array}{ll} \omega^{*}/\omega_{\text{cr}}={\left(\eta_{\text{cr}}/\eta \right)}^{1/2}, & \omega^{*}>\omega_{\text{cr}} \end{array}$$

Eq. 47 can be rewritten as

(49) $$\begin{array}{lll} \eta \omega^{*}/(\eta_{\text{cr}}\omega_{\text{cr}}) & =\omega_{\text{cr}}/\omega^{*}, & \omega^{*}>\omega_{\text{cr}} \end{array}$$

Eqs. 45 and 49 will be discussed comparing experimental data in Section 5.1c.

#### 4.1.e. On the effect of temperature

Now let us consider effect of temperature on the flagellar rotation, concerning the experimental observations listed as (3b), (3c), (3d) in Section 2.2. For comparison, the related quantities are listed below.

$$\begin{array}{llll} \omega =BI_{y}|\Delta \Psi |/(b\eta ), & \omega <\;\omega_{\text{cr}} & & \left(25),(26\right)\\ \eta_{\text{cr}}=2c_{\text{RS}}r_{\text{R}}/b & & & \left(32\right)\\ \omega_{\text{cr}}=B\;I_{y}|\Delta \Psi |/(2c_{\text{RS}}r_{\text{R}}) & & & \left(34\right)\\ \omega *=BI_{y}|\Delta \Psi |/{\left(2c_{\text{RS}}r_{\text{R}}b\eta \right)}^{1/2}, & \omega >\;\omega_{\text{cr}} & & \left(32),(44\right)\\ \omega \;\eta =\eta_{\text{cr}}\;\omega_{\text{cr}}=BI_{y}|\Delta \Psi |/b, & \omega <\;\omega_{\text{cr}} & & \left(32),(34\right)\\ \omega *\eta =BI_{y}\;\eta^{1/2}|\Delta \Psi |/{\left(2c_{\text{RS}}r_{\text{R}}b\right)}^{1/2}, & \omega >\;\omega_{\text{cr}} & & \left(32),(44\right) \end{array}$$

In the flagellar motor, the viscoelasticity of the RS layer seems the most sensitive to temperature variation. The relation *S*_e_(*t*) = *F_y_*(*t*)/*c*_RS_ (Eq. 14) indicates that *c*_RS_ is a constant to show the elastic hardness of the RS layer and should sensitively increase with decreasing temperature, as expected in viscoelastic materials. Accordingly, the quantities depending upon *c*_RS_ in the above list should depend upon temperature significantly. In connection with the experimental observations (3b), (3c), and (3d) in Section 2.2, the followings are mentioned.

(3b) *ωη* does not depend upon *c*_RS_ according to (32), (34).
(3c) *ω*_cr_ is inversely proportional to c_RS_ according to (34).
(3d) ω* and ω*η are proportional to 1/*c*_RS_^1/2^ and hence decreases with increasing *c*_RS_ according to (32), (44).

These conclusions will be discussed in comparison with experimental data in Section 5.1d.

#### 4.1.f. On the energy efficiency

As discussed in Sect 4.1c, the balance of forces can be realized when *D*/*W* < 1 (Eq. 30). The energy difference *W* – *D* is transmitted into the interior of the Rotor as shown by the deformation of the Rotor in Figure 4(b2). In this section, the energy efficiency for the flagellar rotation after the shear forces are transmitted into the RS layer is considered. Let the energy efficiency *e_W_* be defined by

(50) $$\begin{array}{ll} e_{W}=D/W, & \omega <\omega_{\text{cr}} \end{array}$$

From Eqs. 6, 28 and 32, we have

(51) $$\begin{array}{ll} e_{W}=\eta_{\text{cr}}/\eta , & \omega <\omega_{\text{cr}} \end{array}$$

Or from Eq. 3-1d-1, we have

(52) $$\begin{array}{ll} e_{W}=\omega /\omega_{\text{cr}}, & \omega <\omega_{\text{cr}} \end{array}$$

In the case of *ω* > *ω*_cr_, there is no force balance and all the input energy *W* is spent for rotation, and hence the efficiency *e_W_* should be less than

(53) $$\begin{array}{ll} e_{W}*=1, & \omega >\omega_{\text{cr}} \end{array}$$

**Figure 6.**
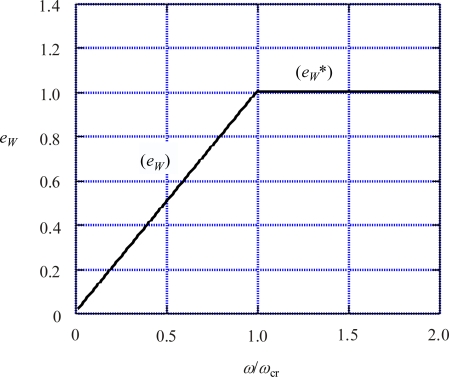
Energy efficiency *e_W_* as a function *ω*/*ω*_cr_. (*e_W_*): *e_W_* when forces are balanced, as calculated by Eq. 52. ( e_W_*): upper limit of *e_W_* given by Eq. 53.

Figure 6 shows the relation Eq. 52 by (*e*_W_) and Eq. 53 by (e_W_*). For *ω* = 0, *e*_W_ = 0 and all *W* is transmitted into the interior of the Rotor. The over-all energy efficiency *e* will reach the maximum at *ω*=*ω*_cr_ where *e_W_* = 1. Let *e* at *ω*_cr_ be denoted as *e*_cr_, then *e* is given by

(54) $$\begin{array}{ll} e=e_{W}e_{\text{cr}}, & \omega <\omega_{\text{cr}} \end{array}$$

(55) $$\begin{array}{ll} e<e_{W}*e_{\text{cr}}, & \omega >\omega_{\text{cr}} \end{array}$$

### 4.2. Effect of reversal of proton passage direction

Several workers examined how the flagellar rotation changes when the direction of the proton movement is reversed by changing the sense of the transmembrane electrochemical potential difference, *Ψ*, as described in detail in Section 5.2. In this section, the effect of reversal of the proton passage direction is discussed based on the model.

The electric field *E_x_* due to the proton pair is determined by the proton position, *z*_p_, as given in Figure 2, irrespective of the direction of their motion. Hence, the shear force *X_y_*(*z*_p_) = *aP*_0*y*_*E_x_*(*z*_p_) (Eq. 1) does not depend upon the direction of proton passage. Therefore, the rotation direction of the Rotor is the same irrespective of the direction of the proton motion, as evident also in Figure 3.

Some readers, however, may feel puzzled about the above conclusion because, if *ν*_p_ is replaced by - *ν*_p_ in *F_y_*(*t*) = *aγP*_0*y*_*E_x_*(*z*_p_)*ν*_p_ (Eq. 11), then *F_y_*(*t*) becomes negative so that the impulse *I_y_* (Eq. 12) and Δ*θ* (Eq. 21) become negative, and thus the rotation direction will be reversed. This conjecture, however, is not correct since the above expression of *F_y_*(*t*) is derived for the case of Δ *Ψ*> 0 Let us derive *F_y_*(*t*) in the case of Δ*Ψ*<0 The position of a proton pair for the reverse passage, is given by

(56) $$z_{\text{p}}=d_{\text{ch}}-\nu_{\text{p}}t$$

Then *E_x_*, which is expressed by *E_x_*(*ν*_p_*t*) as a function of *t* for the normal direction, becomes *E*_x_(*d*_ch_ – *ν*_p_*t*) for the reverse passage. Then d*z*_p_ = − *ν*_p_d*t*, and Eq. 8 becomes

(57) $$\begin{array}{l} I_{W}=\{{\left(a\gamma P_{0y}\right)}^{2}/(2c_{\text{RS}})\}\;\int E_{x}{\left(d_{\text{ch}}-\nu_{\text{p}}t\right)}^{2}\;\text{f(}\nu_{\text{p}}{\text{)}}^{2}\text{dt}\\ \;\;\;\;\;\;\;=\{\text{f(}\nu_{\text{p}}{\text{)}}^{2}/\nu_{\text{p}}\}\{\;{\left(a\gamma P_{0y}\right)}^{2}/(2c_{\text{RS}})\}\;\int E_{x}{\left(z{\;}_{\text{p}}\right)}^{2}\;(-{\text{dz}}_{\text{p}}) \end{array}$$

where the integration by *z*_p_ is done from *d*_ch_ to 0. By changing the integration range from (0 to *d*_ch_) to (*d*_ch_ to 0),

(58) $$I_{W}=\{\text{f(}\nu_{\text{p}}{\text{)}}^{2}/\nu_{\text{p}}\}\{\;\;{\left(a\gamma P_{0y}\right)}^{2}/(2c_{\text{RS}})\}\;\int E_{x}{\left(z{\;}_{\text{p}}\right)}^{2}\;{\text{dz}}_{\text{p}}$$

This equation is the same as Eq. 9, and hence we have f(*ν*_p_)=*γν*_p_, as in Eq, 10, where *ν*_p_ is the absolute value defined by Eq. 3. Therefore, *F_y_*(*t*) becomes

(59) $$\begin{array}{ll} F_{y}(t)=\gamma X_{y}(d_{\text{ch}}-\nu_{\text{p}}t)\nu_{\text{p}}=a\gamma P_{0y}E_{x}(d_{\text{ch}}-\nu_{\text{p}}t)\nu_{\text{p}}, & \text{for}\;\Delta \Psi \;<\;0 \end{array}$$

The integration *I_y_* = ∫*F_y_* (*t*)d*t* is the same for the normal and reversed proton passages since the area under the curve *E_x_*(*t*) and that for *E_x_*(*d*_ch_ – *ν*_p_*t*) are equal. Hence, the rotation velocity *ω* given by Eq. 25 has the same value and the same sense irrespective of the sign of Δ*Ψ*. That is,

(60) ω=C|ΔΨ|

where *C* is the same constant as defined by Eq. 26. Eq. 60 has the same form as Eq. 25, but now it is certain that *C* has the same value for positive and negative. This matter will be discussed in comparison with experimental data in Section 5.2.

### 4.3. Effect of externally applied torque on rotor

Berg and his colleagues [18–20] used the technique of electrorotation to apply torque to *E. coli* bacterial cells tethered to glass coverslips by a single flagellum, as described in more detail in Section 5.3.

Theoretically, the relative motion between the cell and a flagellum is important, and the following discussion is made as if the cell is fixed and a flagellum is rotated by an external force. The externally applied torque is denoted as *T*_app_, which transmits from the flagellum into Mot* through the elasticity of the RS layer, deforming Mot* as shown in Figure 3(b). This deformation will induce an electric field, *E_x_*’ by the inverse piezoelectric effect. The *x* component of the electric field, *E_x_*’, however, does not affect the proton motion along the *z* axis. Although *T*_app_ might produce other components of the field, *E_y_*’ and *E_z_*’ in Mot*, *E_y_*’ does not affect the motion of the proton along the z axis and *E_z_*’ will be compensated as an average by the ions in the outer and inner liquids, since the transmembrane potential tends to keep *E_z_* constant. Accordingly, *T*_app_ does not change the velocity *ν*_p_ of the protons in the channel nor the number *n* of proton pairs passing the membrane per unit time:

(61) $$\nu_{\text{p}}(T_{\text{app}})=\nu_{\text{p}}(0)$$

(62) $$n(T_{\text{app}})=n(0)$$

The torque produced by the cell under the external torque is denoted as *T*_cell_(*T*_app_) and the torque at *T*_app_= 0 as *T*_cell_(0). Since *T*_app_ does not change the way of proton motion, *T*_app_ does not affect the torque produced by the cell:

(63) $$T_{\text{cell}}(T_{\text{app}})=T_{\text{cell}}(0)$$

The rotation velocity produced by the cell itself under the external torque is denoted as *ω*_cell_ (*T*_app_) and the torque at *T*_app_= 0 as *ω*_cell_(0). Eq. 63 means that the deriving force for the cell to produce the flagellar rotation is not affected by *T*_app_. Accordingly, *ω*_cell_ does not depend upon *T*_cell_:

(64) $$\omega_{\text{cell}}(T_{\text{app}})=\omega_{\text{cell}}(0)$$

Effects of *T*_cell_ will be discussed in comparison with experimental observations in Section 5.3.

### 4.4. Switch mechanism for changing rotation direction

Bacteria have a switch to change the direction of flagellar rotation for chemotaxis as described, for instance, in [21]. Presumably a large-scale structural modification is needed to explain the chemotaxis if we assume a direct contact between Mot* and the Rotor. In our model, however, the coupling is intermediated by *P_0y_* and is effected by the shear force *F_y_*(*t*) = *a*γ*P_0y_E_x_*(*z*_p_)*ν*_p_ (Eq. 11). Therefore, the rotation direction can be reversed simply by changing the sign of *P*_0*y*_. More detailed discussion on this matter is given in Section 5.4.

## 5. Comparison of the theoretical predictions with experimental observations

### 5.1. The flagellar rotation velocity and torque

#### 5.1.a. Number of rotation steps per one revolution

At low speeds and high torques, about 1,000 protons are required per revolution of flagellar rotation [1]. In our model, when *ω* < *ω*_cr_, one revolution consists of 2*π*/Δ*θ* steps and Δ*θ* is a constant independent of the transmembrane potential difference Δ*Ψ* as noted at the end of Section 4.1a. Hence one revolution of the flagellar rotation consists of a constant number of steps, irrespective of Δ*Ψ*. As discussed at the end of Section 3.1, Δ*θ* seems large enough to explain 1,000 protons per revolution.

#### 5.1.b. Rotation velocity as a function of the transmembrane potential difference

Fung and Berg [22] drew filamentous cells of *E. coli* into micropipettes, made the transmembrane pH difference zero by using the ionophore gramicidin S, and then energized the system by an external voltage source. They found that torque is proportional to protonmotive force up to –150 mV, and all of the data are consistent with a linear speed-protonmotive force relation. In our model, the transmembrane electrochemical potential difference is defined by Δ*Ψ* = *Ψ*_in_ – *Ψ*_out_ (Eq. 2).

Analogously to this, the electric potential difference Δ*φ* is defined by

(65) $$\Delta \varphi =\varphi_{\text{in}}-\varphi_{\text{out}}$$

**Figure 7.**
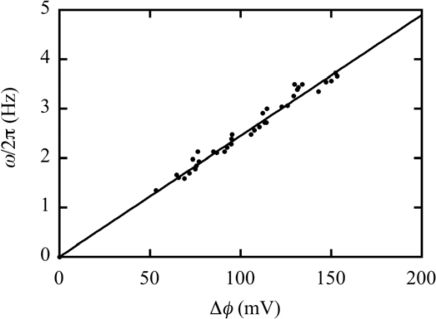
Flagellar rotation velocity *ω*/2*π* as a function of transmembrane electrical potential Δ*φ*. The data points are cited from Figure 4 of the paper of Fung and Berg [22].

The protonmotive force in [22] corresponds to − Δ*φ*. Experimental data in one of the three figures in Figure 4 of [22] are cited by black circles in Figure 7 as a relation between *ω*/2*π* and Δ*φ*. The experiments by Fung and Berg [22] were done for *ω* < *ω*_cr_, and hence we expect the relation *ω*/2*π* = (*C*/2*π|* Δ*φ*| according to Eq. 25. The straight line in Figure 7 was drawn by setting *C*/(2*π* = 0.0245 Hz/mV. The theoretical result is in good agreement with the experimental observations. As Fung and Berg [22] wrote, these results are consistent with a mechanism in which a fixed number of protons, working at unit efficiency, carry the motor in each revolution.

#### 5.1.c. Torque as a function of rotation velocity

**Figure 8.**
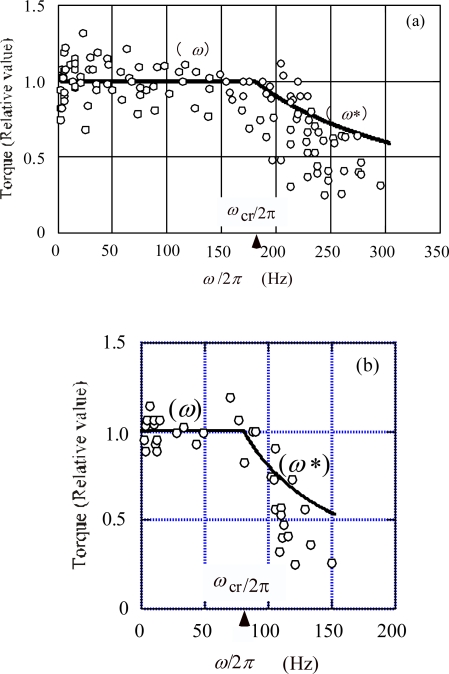
Relative value of torque vs. rotation velocity *ω*/2. Data point are cited from Figure 3 of [1]. (*ω*): Torque when forces are balanced, calculate by Eq. 66. (ω*): Upper limit of torque, calculated by Eq. 67. (a) 23 °C. (b) 16 °C.

The relative values of the torque produced by a flagellum are given as functions of the flagellar rotation velocity in [1, 23]. The data points in Figure 3 of [1] are cited by circles in Figure 8. Since the torque produced by a flagellum is proportional to the viscosity *η*and the rotation velocity *ω*, the relative value of the torque is given by *η*/(*η*_cr_*ω*_cr_) in our model. According to Eq. 46,

(66) $$\begin{array}{ll} \eta \omega /\;(\eta_{\text{cr}}\omega_{\text{cr}})=1, & \omega <\omega_{\text{cr}} \end{array}$$

The straight lines indicated by (*ω*) in Figure 8(a) and (b) correspond to Eq. 66. In our calculation, the critical values *ω*_cr_/(2π) are set equal to 180 Hz and 80 Hz for 23°C and 16°C;, respectively. The data points distribute around the line (*ω*) for *ω*< *ω*_cr_.

In the case of *ω*>*ω*_cr_, where the input energy is fully used for flagellar rotation. According to Eq. 49,

(67) $$\begin{array}{ll} \eta \omega */\;(\eta_{\text{cr}}\omega_{\text{cr}})=\omega_{\text{cr}}/\omega *, & \omega *>\omega_{\text{cr}} \end{array}$$

The curves indicated by (ω*) in Figure 8 were calculated by setting ω* = *ω* in the right-hand side of this equation. Since D* is set equal to the input energy *W* in Section 4-1c,

ω* is the maximum rotation velocity for *ω* > *ω*_cr_,. Therefore, if the observed rotation velocity is denoted as *ω*_obs_,

(68) $$\omega_{\text{obs}}<\omega *$$

(69) $$\begin{array}{ll} \eta \omega_{\text{obs}}/(\eta_{\text{cr}}\omega_{\text{cr}}) & <\eta \omega */(\eta_{\text{cr}}\omega_{\text{cr}}) \end{array}$$

These equations predict that the data points tend to distribute at the left-hand side and below the (ω*) curve, as seen in Figure 8.

#### 5.1.d. On the effect of temperature

The theoretical conclusions about the temperature effects on the flagellar rotation are given in Section 4.1e. They agree with experimental data as described below, with the same item numbers in Sects. 2.2 and 4.1e,

(3b) When *ω* <*ω*_cr_, the torque varies little with temperature because *ωη* does not depend upon *c*_RS_, in agreement with the observation (3b) cited in Section 2.2.
(3c) The critical velocity *ω*_cr_ decreases at lower temperatures because *ω*_cr_ is inversely proportional to c_RS_, in agreement with the experimental results cited in Figure 8 (a) and (b).
(3d) When *ω* <*ω*_cr_, the torque declines more steeply at lower temperature because ω*η is proportional to 1/*c*_RS_^1/2^, in agreement with the experimental results cited in Figures 8 (a) and (b).

### 5.2. Effect of reversal of proton passage direction

**Figure 9.**
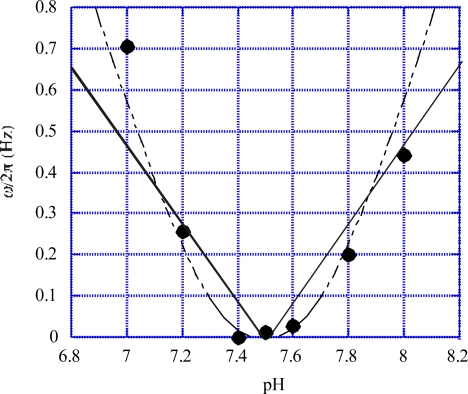
Flagellar rotation velocity *ω*/2*π* as a function of pH of the outer solution. Data points are cited from Figure 5b of Berg *et al.* [25]. The direction of the proton movement is inward for pH < 7.5 and outward for pH > 7.5.

In the experiment [22] mentioned in Section 5-1b, Fung and Berg also tried to examine how the flagellar rotation changes when the direction of the proton movement is reversed by changing the sense of the transmembrane electric potential difference Δ*φ*. It was, however, difficult to reach definite conclusion. They observed that, when the sense of Δ*φ* was reversed, the motors rotated in the same direction for a short time in three of 17 trials, the motor rotated in the reversed direction for a few seconds in five of the trials, the motors stopped immediately in the remaining nine trials. On the other hand, Manson, *et al.* [24] used *Steptococcus* bacteria and examined how the flagellar rotation velocity varies when the direction of proton motion is reversed by changing ionic concentration in the outer solution. They observed that in some specimens, the direction of the rotation remained the same but in other specimens the direction was reversed when the proton movement was reversed. Berg *et al.* [25] carried out more detailed studies of the effects. An example of their results are cited by black circles in Figure 9. They changed pH in the outer solution from 7 to 8, and observed that the rotation velocity decreases, becoming zero around pH = 7.5 and then increases. The direction of the proton passage seems to be reversed at pH = 7.5, but the direction of the rotation remains the same. This feature is consistent with our conclusion *ω* = *C|*Δ*Ψ*| (Eq. 60) where *C* is the same constant for positive and negative Δ*Ψ*. In the experiment cited in Figure 9, the electrochemical potential difference Δ*Ψ* is proportional to (pH – 7.5). The solid lines in Figure 9 show results calculated by

(70) ω/2π=1.88|pH−7.5|

These lines illustrate the theoretical prediction that *ω* is positive irrespectively of the sign of Δ*Ψ*, but the lines do not well represent the distribution of the experimental data. Especially, the rotation velocity is close to zero in the vicinity of pH= 7.5 where the proton pair moves slowly. In the case of large electrical potential difference, however, experimentally observed *ω* are well reproduced by the linear relation *ω* = *C*Δ*φ* as seen in Figure 7. Therefore, Figure 9 indicates that there is a deviation from the linear relation when the proton pair moves slowly, and suggests that some correction is necessary in the postulation that Mot* is a constant shear force transmitter. A possible model would be that the deformation *x_y_* of Mot* has two components, one of which can immediately follow the stress *X_y_* and the other responses against *X_y_* with significant delay. In [2] a model was proposed assuming such a response delay. It was postulated that there is a large delay in the deformation *x_y_* against the stress *X_y_* in Mot* for not-very-slow proton passage, but *x_y_* can quasi-stationarily follow *X_y_* for slow proton passage. Then the shear force integral *I_y_* (Eq. 12) becomes practically constant for usual proton passage but approach to zero for slow proton passage. The calculated values of *ω*/2*π*, which are cited from Figure 11 of [2], are given by the dashed curve in Figure 9, which well reproduces the general tendency of the experimental data.

Invariance of the rotation direction similar to the data shown in Figure 9 was observed in some bacterial strains, but the rotation direction was reversed in some other strains when the direction of the proton passage is reversed [25]. These indefinite results were attributed to the sensitivity of the switch mechanism to ionic circumstances in [25]. More experimental studies seem desired. Sometimes the invariance of the rotation direction was doubted in relation with a possibility of perpetual flagellar rotation caused by Brownian motion of protons in the channel. A comment is given about this problem in Sect. 6.1.

### 5.3. Effect of externally applied torque on rotor

**Figure 10.**
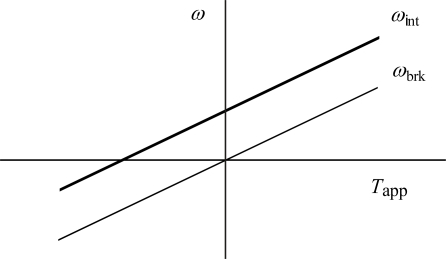
Schematic illustration of the effect of the externally applied torque *T*_app_ on the cell rotation velocity *ω* for E. coli cell [19, 20]. The cell is tethered by a single flagellum and the cell body is rotated electrically. *ω*_int_ and *ω*_brk_ are rotation velocities of the cell intact and the cell broken, respectively.

As mentioned in Section 4.3, Berg and his colleagues [18–20] used the technique of electrorotation to apply torque to cells of the bacterium *E. coli* tethered to glass coverslips by a single flagellum. Cells were driven to rotate either forward or backward. Here forward means the rotation driven by the flagellar motor itself. They used the intact cells which can normally rotate the flagellum (called motor intact) and those which lost the ability to rotate actively the flagellum (called motor broken). Berg and Turner [18] observed a barrier to backward rotation for the motor intact but later Berry and Berg [19, 20] found that it was an artifact and showed that the relation between rotation velocity and torque is approximately linear in the range over 100 Hz in either direction and parallel to the relation of the cell broken. Let us denote the rotation velocities of the intact and broken cells as *ω*_int_ and *ω*_brk_, respectively. The externally applied torque is expressed by *T*_app_ as in Section 4.3. With these symbols, the experimental results by Berry and Berg [19, 20] are schematically illustrated by the two lines *ω*_int_ and *ω*_brk_ in Figure 10.

The rotation velocity *ω*_brk_ is proportional to *T*_app_:

(71) $$\omega_{\text{brk}}(T_{\text{app}})=qT_{\text{app}}$$

where *q* is a constant. In Section 4.3, the rotation velocity produced by the cell itself under the torque is denoted as *ω*_brk_(*T*_app_), and the relation (Eq. 64) is derived as

(72) $$\omega_{\text{cell}}(T_{\text{app}})=\omega_{\text{cell}}(0)$$

This means that ability of the cell to produce the flagellar rotation is not affected by *T*_app_ and hence it is reasonable to expect the additive relation between *ω*_brk_(*T*_app_) and *ω*_cell_(0) for the intact cell. Thus, considering Eq. 71, we have

(73) $$\omega_{\text{int}}(T_{\text{app}})=qT_{\text{app}}+\omega_{\text{cell}}(0)$$

That is, the model gives parallel relation between *ω*_int_(*T*_app_) and *ω*_brk_(*T*_app_), in agreement with experimental observation illustrated in Figure 10. As mentioned above, experimentally the parallel relations in Figure 10 holds from −100 Hz to +100 Hz. This implies that *ω*_cell_(*T*_app_) = *ω*_cell_(0) (Eq. 72) holds and the proton motion is not affected even for negative *T*_app_. Therefore, negative *T*_app_ does not pump out protons unlike the case of F_0_F_2_-ATP synthase (cf. Section 6.2).

### 5.4. Switch mechanism for changing rotation direction

Bacteria have a switch to change the direction of flagellar rotation for chemotaxis. For details of the switch, readers may refer, for instance, to [21].

As described in Section 3.1, the MotA complex consists of 4 copies and MotB complex consists of 2 copies in Mot*. All the copies are expected to have permanent electric dipoles. We defined the polarization corresponding to the vector sum of their dipoles as Mot* polarization, and denoted its *y* component as *P*_0*y*_. As mentioned in Section 4.4, the flagellar rotation direction can be switched by changing the sign of the polarization *P*_0*y*_ in our model. If the dipoles of copy molecules have a tendency to be approximately antiparallel to each other, their vector sum, and thus Mot* polarization and *P*_0*y*_, will be sensitive to change in structure and orientation of the copy molecules. Then, relatively small structural or orientation changes in the copy molecules can cause change of the sign of *P*_0*y*_. It is a possibility that Stator can assume two stable structures having positive and negative *P*_0*y*_ and the chemotaxis system determines which of the two is realized through the chemical information flow as illustrated in Figure 2 of [21]. Then the shear forces *F_y_*(*t*) = *aγP_0y_E_x_*(*z*_p_)*ν*_p_ (Eq. 11) simultaneously change their sign in all Mot*s of Stator and the flagellar rotation is reversed.

## 6. Discussion

### 6.1. Summary and discussion

As mentioned in Section 1, protein molecules are structurally polar and thus piezoelectric, and there should be interaction between an electric field and their mechanical deformation. Hence theoretically the flagellar rotary motor should be treated as a four-variable system, i. e., as a system in which strain, stress, electric field and polarization are interacting with each other. Our model is constructed based on this viewpoint and succeeds in explaining various experimental data. The viewpoint of four-variable system was applied to discuss the muscle contraction mechanism and successful to explain experimental observations [26]. It is assumed that Mot* has a permanent polarization and acts as a shear force generator. The shear force is transmitted to the Rotor through the viscoelastic RS layer. Experimental observations listed as items (1) ~ (6) in Section 2.2 are explained by the model. The results are summarized below with the same item numbers.

(1) When the flagellar rotation velocity *ω* is smaller than the critical velocity *ω*_cr_ , one revolution of the flagellar rotation consists of a constant number of rotation steps as proved at the end of Section 5.1a.
(2) When *ω*<*ω*_cr_, the rotational velocity *ω* is proportional to the transmembrane electric potential difference Δ*Ψ* as given by Eq. 25 and shown in Figure 7, in agreement with experimental observations.
(3a) As indicated by the lines and curves in Figure 8, the torque exerted on the flagella by the cell is independent of the flagellar rotation velocity *ω* and remains constant when *ω* < *ω*_cr_, and then sharply decreases above *ω*_cr_, in agreement with the experimental data.
(3b) When *ω*< *ω*_cr_, the torque is expected to vary little with temperature, as discussed in Section 5.1c, in agreement with experimental observations.
(3c) The critical velocity *ω*_cr_ shifts to lower speeds at lower temperatures as discussed in Section 5.1c (cf. Figure 8(a) and (b)).
(3d) When *ω*>*ω*_cr_, the torque declines more steeply at lower temperatures as discussed in Section 5.1c (cf. Figure 8(a) and (b)).
(4) The model predicts that the flagella rotate in the same direction when direction of the proton passage is reversed, as discussed in Section 4.2 and 5.2 (cf. Figure 9).
(5) The cell produces constant torque for the flagellum even when the cell is rotated relative to the flagellum by external forces, as discussed in Sects. 4.3 and 5.3 (cf. Figure 10).
(6) It is possible that the cell reverses the sense of the flagelllar rotation with the same absolute value of velocity if the direction of *P*_0*y*_ is changeable by chemical modification of Mot*, as discussed in Sects. 4. 4 and 5. 4.

As mentioned in Section 2.1, each Mot* has two proton channels [6], but two protons seem to pass practically simultaneously through the two channels [6]. For simplicity, we have supposed that the two protons simultaneously pass through the combined single channel as shown in Figure 1. It seems plausible that Coulomb field produced by one proton in one channel is very strong at the other channel as suggested by Figure 2, and largely deforms the potential barrier for the proton in the other channel so as to stimulate simultaneous passage of the proton pair.

Sowa *et al.* [27] observed 26 steps per one flagellar revolution. It is an interesting result. As mentioned in Section 1, however, many (about 1000) protons are needed for one flagellar revolution, and our present viewpoint is that the flagellar rotation is a more physical rather than chemically specific process. From this viewpoint, the observed 26 steps seem to relate with rugged potential distribution for the flagellar rotation which is caused by the structural periodicity of the ring of FliG proteins.

A question arose whether perpetual flagellar rotation occurs by Brownian motion of the protons in channel if the reversal of proton passage causes the flagellar rotation in the same direction. An answer can be found in the experimental data in Figure 9, where the three data points indicate that *ω* is almost zero when pH is close to 7.5, illustrating that the flagella rotation does not occur when proton-motive force is small and protons move slowly. Theoretically, there seem to be two origins causing this observation. (1) Generally the viscoelastic medium loses its ability to transmit the shear stress when the shear changes very slowly with time, as described in text books (For instance, [17]). (2) When Mot* deformation is faster than rotor reaction, then the shear force *X_y_* is released mainly within Mot* and RS layer interact with Mot* through strain rather than stress. Case (2) was treated mathematically with a finite time constant of Mot* deformation in [2]. The time constant was set so that almost all shear stress is released by deformation of Mot* for slow proton passage. The calculation results in [2] are cited by the dotted curve in Figure 9, which has zero tangent at pH = 7.5. Thermal motion of protons in channels seems slow in channels so that it is decoupled from the motion of the flagellar rotor. Accordingly, thermal fluctuation of the proton does not cause the perpetual flagellar rotation.

### 6.2. Comparison of the flagellar motor with the F_O_F_1_-ATPase motor

Another example of biological rotary motors is F_0_F_2_-ATP synthase. Protons pass through the channels due to the transmembrane electrochemical potential difference and rotates the rotor which consists of F_0_c ring and γ protein. The reversed rotation of the rotor by ATP hydrolysis pumps out protons through the channels (for instance, cf. [28]). On the other hand, in the case of the bacterial flagellar motors there is no observation of proton pumping.

From the structural viewpoint, the motor of F_0_F_2_-ATP synthase is quite different from the bacterial flagellar motors. The diameter of the flagellar motor is about 45 nm while that of F_0_F_2_-ATP synthase is about 10 nm [29]. The diameter of a *γ* protein molecule which rotates in F_1_ is about 2 nm, and has binding sites at every 120°. Thus there seems to be a device to prevent backward motion like a microscopic ratchet. The *γ* protein molecule is surrounded by about 10 F_0_c-subunits [28]. About 10 protons can cause 360° rotation of the rotor. This means that one proton passage per F_0_c-subunit is large enough to cause one revolution of the rotor. Presumably each hopping step of the proton motion is connected with each sep of chemical relation, and such models as proposed by [30, 31] seem close to reality. In these models, reversibility of the motor is possible: flow-in protons cause ATP synthesis and ATP dehydration causes the proton pumping.

In the case of the flagellar rotary motor, the stator consists of about eight pairs of Mot* (Figure 1) and about 1000 protons (500 proton pairs) are needed for one revolution of the rotor [1]. Hence 500/8 = 63 relative positions exist for the proton pair per Mot*. It seems difficult to suppose 63 chemical reaction sites for proton pair per Mot*, and it seem plausible that the flagellum rotates by a chemically non-specific force such as proposed in our model.

## Figures and Tables

**Figure 11.**
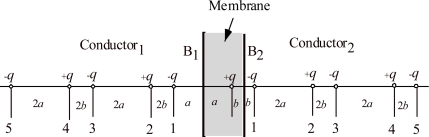
Calculation of *E_x_* presented in Figure 2. Illustration of the method of images.

## Appendix

### Calculation of the electric field produced by the proton pair in dielectric membrane

In our model, the flagellar rotation is induced originally by the shear stress *X_y_*(*z*_p_) = *aP_0y_E_x_*(*z*_p_) (Eq. 1) produced in Mot*. In Section 3.1, Figure 2 is used to give a rough idea on the magnitude of the electric field *E_x_*(*z*_p_) in Mot*. The values in Figure 2 are calculated with a dielectric membrane, and some details of the calculation are given in this Appendix.

Let us consider a uniform dielectric membrane sandwiched by conductive ionic solutions. The proton pair 2e pass through the membrane along the *z* axis that is perpendicular to the membrane. The coordinate of the proton pair is denoted as *z*_p_ which changes from 0 to *d*_ch_. The membrane extends infinitely to the *x* and *y* directions with surface boundaries indicated as B_1_ and B_2_, as shown in Figure 11. The membrane is sandwiched by Conductor_1_ and Conductor_2_. The membrane thickness *d*_ch_ is set equal to *d*_ch_ = 7 nm and its relative dielectric constant *ɛ* = 2. Calculation was carried out based upon by the method of images (for instance, cf. [32]). The real electric charge is shown by +q in the membrane. To use the method of images, the conductors are replaced by dielectric materials of dielectric constant *ɛ* and imaginary electric charges +*q* and –*q* are put along the *z* axis outside the membrane as shown in Figure 11. The imaginary charges are arranged antisymmetric both with respect to B_1_ and B_2_, so that the electric fields on B_1_ and B_2_ are perpendicular to the boundaries, satisfying the boundary conditions. The electric field in the membrane is obtained for these charge distribution.

Then, a cylinder of radius *R* is considered in the membrane around *z* axis. The distance between the channel and the Rotor surface seems unknown, and *R* was set equal to 2.5 nm in the calculation. The volume-average of the *x* component of the electric field was calculated for the half of the cylinder in positive *x*. The volume-averaged *x* component is given as *E_x_* in Figure 2.
